# Economic evaluations of alcohol prevention interventions: Is the evidence sufficient? A review of methodological challenges

**DOI:** 10.1016/j.healthpol.2017.10.003

**Published:** 2017-12

**Authors:** Sarah R. Hill, Luke Vale, David Hunter, Emily Henderson, Yemi Oluboyede

**Affiliations:** a Institute of Health and Society, Newcastle University, Baddiley-Clark Building, Richardson Road, Newcastle upon-Tyne, NE2 4AX, UK; b Centre for Public Policy and Health, School of Medicine, Pharmacy & Health, Wolfson Research Institute, Durham University, Queen’s Campus, Stockton-on-Tees, TS17 6BH, UK; c Fuse, UKCRC Centre for Translational Research in Public Health, Newcastle upon-Tyne, UK

**Keywords:** Economic evaluation, Public health, Alcohol interventions, Priority-setting, Health economics, Methodological challenges

## Abstract

Public health interventions have unique characteristics compared to health technologies, which present additional challenges for economic evaluation (EE). High quality EEs that are able to address the particular methodological challenges are important for public health decision-makers. In England, they are even more pertinent given the transition of public health responsibilities in 2013 from the National Health Service to local government authorities where new agents are shaping policy decisions.

Addressing alcohol misuse is a globally prioritised public health issue. This article provides a systematic review of EE and priority-setting studies for interventions to prevent and reduce alcohol misuse published internationally over the past decade (2006–2016). This review appraises the EE and priority-setting evidence to establish whether it is sufficient to meet the informational needs of public health decision-makers.

619 studies were identified via database searches. 7 additional studies were identified via hand searching journals, grey literature and reference lists. 27 met inclusion criteria. Methods identified included cost-utility analysis (18), cost-effectiveness analysis (6), cost-benefit analysis (CBA) (1), cost-consequence analysis (CCA) (1) and return-on-investment (1). The review identified a lack of consideration of methodological challenges associated with evaluating public health interventions and limited use of methods such as CBA and CCA which have been recommended as potentially useful for EE in public health. No studies using other specific priority-setting tools were identified.

## Highlights

• There are few economic evaluations of alcohol prevention interventions.
• Consideration of impacts beyond an individual’s health in evaluations is limited.
• No published studies using other priority-setting methods in the alcohol area.
• Consideration of wider societal perspectives and health inequalities is minimal.
• Including inter-sectoral costs and consequences in evaluations is challenging.

## 1 Introduction

An increasing need for high quality economic evaluations of public health interventions is recognised and has been documented by academics and other commentators [1], [2], [3], [4]. Characteristics unique to public health, compared to healthcare technologies, present additional challenges to the evaluation of public health interventions. The reach of public health intervention consequences is much broader than healthcare technologies where commonly an individual beneficiary can be identified and the outcome of interest is health maximisation. The time lag between intervention and effect can also be considerably longer in public health, compared to a health technology, where the aim is often to prevent future morbidity; this poses evaluative challenges in the form of discounting future costs and benefits and modelling of longer-term effects. Costs incurred and benefits experienced in the present are generally valued greater than those in the future, therefore when modelling interventions with long-term costs and benefits a discount rate should be applied to reflect the reduced value of future costs and benefits to a decision-maker today; the discount rate applied may affect the outcome of an economic evaluation, therefore must be chosen carefully.

The reported paucity of high quality economic evaluations in public health may in part be due to a lack of consensual methodological guidance on the conduct of economic evaluations in this area [5], [6], [7]. The unique challenges for evaluating public health perhaps make the use of common evaluative methods, for which guidelines exist for their conduct and reporting [8], [9], [10], such as cost-utility analysis, insufficient for the task. Consequently, alternative methods for which there is less established guidance in health care may need to be used resulting in public health economists using a heterogeneity of evaluative methods [11]. The lack of guidance may also be behind the poor quality of many evaluations that have been published [12] since a lack of consensus over the methods to use and the costs and consequences to include [13], [14] could contribute to results of varying quality. This lack of methodological consensus prevents easy comparison between different public health interventions by decision-makers and is unhelpful for researchers conducting such evaluations.

Several reviews have been conducted looking at methods of economic evaluation in public health [2], [3], [5]. Edwards et al. [5] conducted a comprehensive review of guidance documents to identify potential gaps in instruction for public health evaluations, however this review did not look at how evaluations are actually being conducted in practice. Owen et al. [2] focussed on the cost-effectiveness of published National Institute of Health and Care Excellence (NICE) public health guidance which limited the review to consider only evaluations that have been used by NICE. Weatherly et al. [3] identified evaluative challenges via a review of evaluations that have been conducted on a range of public health interventions. Whilst this review was comprehensive, it is limited to evaluations published between the years 2000–2005 thus will not have captured evaluations that have been conducted since recent guidance on public health economics has been released. For example, the majority of the guidance documents identified by Edwards et al. [5] was published after 2005.

In the UK, English public health responsibilities were transferred to local authorities in 2013. The result of this move is that policy decisions are being shaped and influenced by new agents, such as locally elected politicians. How prioritization decisions will be made, using which approaches, in this new context merits scrutiny [15]. Alongside this shift in the public health context in England and to address the lack of methodological guidance, NICE published updated guidance on the evaluation of public health interventions [16]. It recommended that the wider societal and environmental costs and benefits of public health interventions should be considered via greater use of methods such as cost-benefit analysis (CBA) and cost-consequence analysis (CCA) (see NICE glossary [17] p.216 for definitions of CBA and CCA).

This study will build on the evidence provided by existing reviews and look at economic evaluations of public health interventions around alcohol prevention, a globally prioritised issue [18], [19], [20]. The review will identify evaluations from 2006 to 2016 to capture evidence that has been published since Weatherly et al. [3] conducted their study and since recent recommendations for methods of evaluating public health interventions have been published [5]. This review will also look at methods of priority-setting, such as option appraisal, (social) return-on-investment (ROI/SROI), programme budgeting and marginal analysis (PBMA) and multi-criteria decision analysis (MCDA), to help meet the needs of new public health decision-makers [15], [21].

PBMA and MCDA are both systematic processes to aid resource prioritization decisions which involve assessing the available options against a set of criteria (see [22], [23] for detailed explanations). SROI studies demonstrate the return on an investment considering a wider remit than standard ROI as the benefits to society are also included. Public Health England [24] recently recommended this tool for use by commissioners in drug and alcohol treatment areas, however it may also prove beneficial for investment decisions around non-treatment related alcohol prevention interventions.

### 1.1 Aims and objectives

This review aims to identify the methods of evaluation being used to appraise interventions to prevent excessive alcohol consumption and establish whether published studies provide sufficient information to meet the requirements of public health decision-makers. Particular focus will be given to CBA and CCA, as recommended by NICE, as well as prioritization tools such as PBMA and MCDA. Specific elements of evaluation, inspired by the work of Weatherly et al. [3] and guidance on methods of public health appraisal [25], will provide a focus for the literature search; guidance specific to the United States has also been published [26] although this review will be focusing on the more recent guidance produced by NICE. Such elements include: measurement of outcomes, especially long-term outcomes; study perspectives; apportioning inter-sectoral costs and consequences; and health-equity considerations.

## 2 Methods

A systematic literature review was carried out by one researcher with assistance from an information specialist and two other researchers who co-screened records and verified data extraction.

Details of the protocol for this systematic review were registered on PROSPERO and can be accessed at www.crd.york.ac.uk/PROSPERO/display_record.asp?ID=CRD42016039063.

### 2.1 Literature search

A literature search (see Table 1 for main search terms) was carried out in the NHS Economic Evaluation Database (NHS EED) and Scopus for the time period January 2006 − May 2016. NHS EED is a database of economic evaluations which have been identified through a systematic search of the literature by the Centre for Research and Dissemination (CRD) in York. Since methods of priority-setting are not included in the search strategy used by the CRD, an additional search was conducted in Scopus, limited to the health and social sciences sector, to capture additional priority-setting studies.

**Table 1:** Key search terms used in literature searches.

Search terms^a^
Economics
Health Economics
exp Economic Evaluation
exp Health Care Cost
exp “costs and cost analysis”
economic$ or cost or costs or costly or costing or price or prices or pricing or pharmacoeconomic$
value for money
budget$
(MCDA or PBMA)
“option appraisal”
“multi$ criteria decision analys$"
“program$ budget$ marginal analys$”
(Priority?setting adj2 method$).
“social return on investment”
(SROI or ROI).
“return on investment”
(intoxica$ or beer or wine)
*drinking behaviour
Alcoholic Beverages
*Binge Drinking
Alcohol Drinking
*Alcoholism
“Drink$ behavio$" or “binge drink$"
Alcohol$ adj2 (“use disorder$" or abuse or beverage$ or addiction$ or consumption or drink$))

a Exact search strategy differed between databases therefore full strategy not represented here.

The NHS EED database ceased to be updated from 31st December 2014, therefore a further search was conducted on the databases searched by CRD (Medline, Embase, psychINFO and Cinahl) using a strategy based on that used by CRD in order to capture economic evaluations published between January 2015 and May 2016 (Full search strategies for each database can be viewed in Appendix A of the supplementary material). A hand search of relevant health economics and economics journals was also conducted alongside reference and citation searches of included items.

Grey literature, in the form of public health/health economic conference abstracts, OpenGrey, governmental departments and voluntary organisations’ websites and dissertation and thesis abstracts via ProQuest, was also searched for additional records.

### 2.2 Eligibility criteria

Economic evaluations, defined as the comparative analysis of alternatives with respect to their associated costs and health consequences, or a method of priority-setting defined as a systematic method of deciding where investments (and disinvestments) should be made to best meet the needs of communities, were included for review. Studies were included if they evaluated a public health intervention focussed on preventing alcohol misuse or reducing excessive alcohol consumption. Interventions to prevent alcohol misuse may be targeted at populations at-risk of drinking to prevent future excessive consumption of alcohol or may be targeted at those who drink harmful levels of alcohol. The precise classification of “harmful” alcohol consumption can be defined using a variety of different measures and may differ internationally; therefore no specific definition will be provided as inclusion criteria in order to allow for the inclusion of a range of studies internationally and in an array of settings. Examples of such preventive interventions are: national policies such as minimum unit pricing of alcohol or targeted interventions such as brief interventions in schools or hospitals. Studies were not excluded by country of origin provided they were published in English.

Evaluations of pharmacotherapies were excluded as these would fall within Health Technology Assessment (HTA) rather than public health evaluation, which often uses far more prescriptive methodology, with the dominant method being cost-utility analysis [27]. Evaluations of treatments for alcohol dependency, e.g. detoxification or rehabilitation, were also excluded as these would not be considered preventive; treatments which are part of a preventive regime, such as screening and brief intervention for non-treatment seeking individuals, were included. Evaluations of interventions to prevent harm or injury caused as a result of alcohol consumption, such as traffic accidents resulting from drunk-driving or risky sexual behaviour, were excluded unless the intervention specifically focussed on reducing alcohol consumption as a primary objective.

### 2.3 Data extraction

Studies selected for full-text screen were downloaded into an Endnote X7 literature database and reviewed against the inclusion criteria. A data extraction form was developed based on items used by Drummond et al. [28] in their review which considered similar data (see Appendix B in the supplementary material for an example data extraction). We then added items relevant to this study specifically, such as methods of priority-setting adopted. The form was piloted using the first five studies, reviewed and relevant amendments made. The finalised data for extraction included: intervention and comparators, type of study (randomised controlled trial, non-randomised study or modelling study), population and setting, study length, time-horizon for analysis, perspective, method of economic evaluation or priority-setting, extrapolation of data, reported justification, strengths and weaknesses of methods used, outcomes measured, types of costs included, whether productivity changes were accounted for and equity considerations.

## 3 Results

**Fig. 1:**
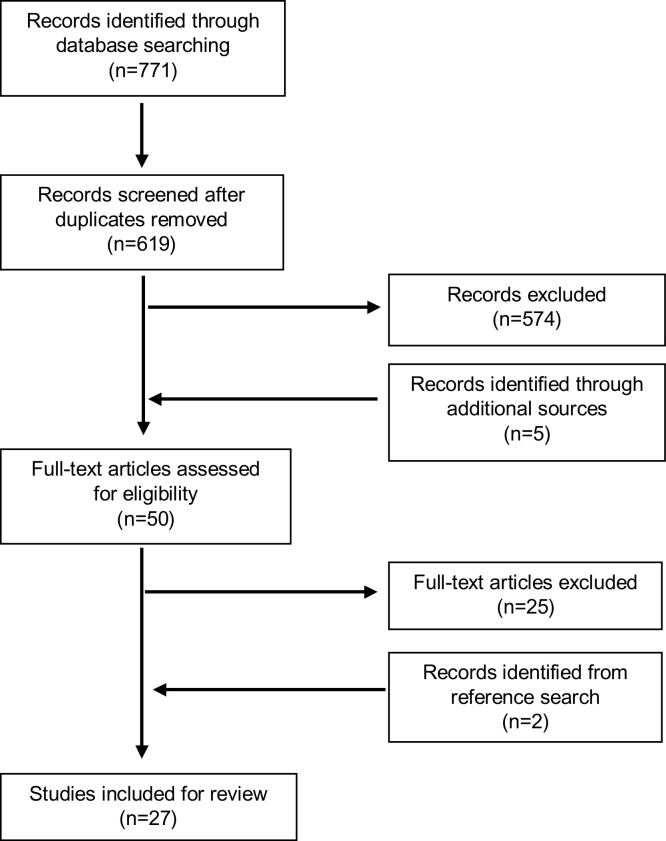
Prisma flow diagram.

Six hundred and nineteen studies were retrieved from the database search after deduplication. Initial screening by two researchers (SH and YO) identified 45 studies for full text screening. A further 5 studies were identified from hand-searching key journals and grey literature. Twenty-five studies were selected for inclusion in the review. A hand-search of references and citations of the included studies identified a further two items for review. A total of 27 items were included for review [29], [30], [31], [32], [33], [34], [35], [36], [39], [40], [41], [42], [43], [44], [53], [54], [57], [65], [68], [70], [75], [76], [77], [78], [79], [80], [81] (Fig. 1).

The included studies were published between years 2006 and 2015, publication frequency was distributed fairly evenly across the time period. Studies were conducted in the following countries: USA (n = 8), UK (n = 5), Australia (n = 5), Netherlands (n = 3), Denmark (n = 2), Estonia (n = 1), Italy (n = 1), Sweden (n = 1) and multi-national (OECD) (n = 1).

The most common primary method of economic evaluation used was cost-utility analysis (CUA) (n = 18) followed by cost-effectiveness analysis (CEA) (n = 6), CCA (n = 1) and CBA (n = 1). Six studies conducted multiple evaluations; four conducted a CEA as a secondary analysis, one a ROI and one a cost-analysis.

One ROI study was identified. No studies using other priority-setting methods were identified (Table 2).

**Table 2:** Overview of studies included in the review and key details, numbered.

Study No.	Author	Year	Country of study	Title	Intervention	Comparator	Method of analysis^a^
1	Angus, C. et al.	2014	Italy	Cost-effectiveness of a programme of screening and brief interventions for alcohol in primary care in Italy	Alcohol screening and brief interventions (SBI) in primary care. Two screening options:	No intervention	CUA
					• At next general practitioner (GP) visit • At registration with GP		
2	Barbosa, C. et al.	2015	United States	The cost-effectiveness of alcohol screening, brief intervention and referral to treatment in emergency and outpatient medical settings	Screening and brief intervention with feedback, intervention or treatment (SBIRT) to all individuals presenting for emergency care but not specifically seeking treatment for substance abuse.	SBIRT in outpatient care	CUA & CEA
3	Barrett, B. et al.	2006	United Kingdom	Cost-effectiveness of screening and referral to an alcohol health worker in alcohol misusing patients attending an A&E department	Opportunistic identification of hazardous drinking and referral to an alcohol health worker. Adults attending A&E selectively screened for alcohol misuse	Information only	CEA
4	Byrnes, J.M. et al.	2010	Australia	Cost-effectiveness of volumetric alcohol taxation in Australia	Volumetric alcohol taxation. Three tax scenarios modelled:	Current tax policy	CUA
					• Maintain current deadweight loss of taxation • Maintain existing taxation revenue • Equate to existing rate for spirits		
5	Cobiac, L. et al.	2009	Australia	Cost-effectiveness of interventions to prevent alcohol-related disease and injury in Australia	Eight interventions evaluated (incl. comparator):	Current Practice (i.e. random breath testing)	CUA
					• Advertising bans • Licensing controls restricting opening hours • Brief intervention • Residential treatment • Raise minimum drinking age • Drink driving campaigns • Volumetric taxation		
6	Cowell, A.J. et al.	2012	United States	Cost-effectiveness analysis of motivational interviewing with feedback to reduce drinking among a sample of college students	Motivational interviewing, assessment and feedback. Four intervention conditions evaluated:	Incremental comparison of all interventions	CEA
					• Assessment only (AO) • Motivational interviewing (MI) • Feedback (FB) • Motivational interviewing and feedback (MIFB)		
7	Crawford, M.J. et al.	2014	United Kingdom	The clinical and cost-effectiveness of brief advice for excessive alcohol consumption among people attending sexual health clinics: a randomised controlled trial	Opportunistic brief advice for excessive alcohol use among people who attend sexual health clinics	General health information leaflet provided	CUA
8	Drummond, C. et al.	2009	United Kingdom	Effectiveness and cost-effectiveness of a stepped care intervention for alcohol use disorders in primary care: pilot study	Stepped care alcohol intervention	Brief intervention	CUA
9	Havard, A. et al.	2012	Australia	Randomized Controlled Trial of Mailed Personalized Feedback for Problem Drinkers in the Emergency Department: The Short-Term Impact	Mailed personalised feedback after screening in the emergency department	No mailed feedback	CEA
10	Holm, A.L. et al. (a)	2014	Denmark	Cost-effectiveness of changes in alcohol taxation in Denmark: a modelling study	Alcohol taxation. Three tax scenarios modelled in:	Current level of taxation	CUA
					• 20% increase in tax • 100% increase in tax • 10% decrease in tax		
11	Holm, A.L. et al. (b)	2014	Denmark	Cost-Effectiveness of Preventive Interventions to Reduce Alcohol Consumption in Denmark	Six interventions to reduce alcohol consumption:	Current practice for each intervention	CUA
					• 30% increase in tax • Raise minimum drinking age • Advertising bans • Limited retail sale hours • Brief telephone intervention • Longer intervention in prevention centres		
12	Ingels, J.B. et al.	2013	United States	Cost-effectiveness of the strong African American families-teen programme: 1-year follow-up	Family skills training to reduce substance abuse in African American adolescents	Attention control intervention	CEA
13	Kapoor, A. et al.	2009	United States	Cost-effectiveness of screening for unhealthy alcohol use with% carbohydrate deficient transferrin: results from a literature-based decision analytic computer model	Screening for unhealthy alcohol abuse using a%CDT test. Four scenarios modelled:	Incremental comparison of all interventions	CUA
					• AUDIT questionnaire only • %CDT test only • AUDIT questionnaire followed by%CDT test • No screening		
14	Lai, T. et al.	2007	Estonia	Costs, health effects and cost-effectiveness of alcohol and tobacco control strategies in Estonia	Five different strategies to reduce alcohol consumption:	No intervention and incremental comparison of all interventions/interventions in combination	CUA
					• Excise tax • Reduced access to alcoholic beverage retail outlets • Advertising ban • Roadside breath-testing • Brief intervention in primary care		
15	Mansdotter, A.M. et al.	2007	Sweden	A cost-effectiveness analysis of alcohol prevention targeting licensed premises	A three component intervention: community mobilisation, a two-day responsible beverage service training course for servers, doormen, and restaurant owners and increased enforcement of alcohol laws	No direct comparator	CCA & CA
16	Miller, T.R. et al.	2007	United States	Effectiveness and benefit-cost of peer-based workplace substance abuse prevention coupled with random testing	Peer-based workplace substance abuse prevention (PeerCare) with random alcohol testing	PeerCare programme without alcohol testing	ROI
17	Miller, T.R. & Hendrie, D.	2008	United States	Substance Abuse Prevention Dollars and Cents: A Cost-Benefit Analysis	School based interventions to prevent substance abuse (alcohol and drugs and tobacco).	No direct comparator	CBA
18	Navarro, H.J. et al.	2011	Australia	The potential cost-effectiveness of general practitioner delivered brief intervention for alcohol misuse: evidence from rural Australia	Screening and brief intervention (SBI) for risky drinking by a general practitioner. Three levels of SBI increase modelled:	Current practice	CEA
					• 10% increase • 20% increase • 100% increase		
19	Neighbors, C.J. et al.	2010	United States	Cost-effectiveness of a motivational intervention for alcohol-involved youth in a hospital emergency department	Motivational interviewing amongst youths admitted to the emergency department of a trauma ward for drinking-related injuries	Standard care	CUA & CEA
20	Purshouse, R.C. et al.	2013	United Kingdom	Modelling the cost-effectiveness of alcohol screening and brief interventions in primary care in England	Alcohol screening and brief interventions (SBI) in primary care. Two screening options:	Current practice	CUA
					• At next general practitioner (GP) visit • At registration with GP		
21	Sassi, F. et al.	2015	Multi-national	Health and economic impacts of key alcohol policy options	Multiple interventions examined:	Interventions compared to each other intervention and to other possible uses of health funds	CUA
					• Brief interventions • Tax increases • Drink-drive regulation enforcement (breath-testing) • Opening hours regulation • Treatment of dependence • Advertising regulation • Minimum price • Workplace interventions • School-based programmes		
22	Shanahan, M. et al.	2006	Australia	Modelling the costs and outcomes of changing rates of screening for alcohol misuse by GPs in the Australian context	Four strategies to improve screening and BI rates for Australian GPs:	Current practice	CEA
					• Academic detailing • Interactive continuing medical education • Computerised reminders • Target payments		
23	Smit, F. et al.	2011	Netherlands	Modelling the cost-effectiveness of health care systems for alcohol use disorders: how implementation of eHealth interventions improves cost-effectiveness	eHealth interventions augmenting national health care system. Two strategies modelled:	Current practice (no eHealth intervention)	CUA & ROI
					• Adding eHealth systems to conventional care • 50% substitution of conventional face-to-face with eHealth interventions		
24	Solberg, L.I. et al.	2008	United States	Primary care intervention to reduce alcohol misuse: ranking its health impact and cost effectiveness	Screening and brief intervention in primary care	No screening	CUA
25	Tariq, L. et al.	2009	Netherlands	Cost-effectiveness of an opportunistic screening programme and brief intervention for excessive alcohol use in primary care	Screening and brief intervention in primary care	Current practice (no SBI)	CUA & CEA
26	van den Berg, M. et al.	2008	Netherlands	The cost-effectiveness of increasing alcohol taxes: a modelling study	Two alcohol tax increase scenarios:	Current practice	CUA & CEA
					• Increase tax on beer only (Dutch scenario) • Raise taxes to on beer, wine and spirits (Swedish scenario)		
27	Watson, J. et al.	2013	United Kingdom	AESOPS: a randomised controlled trial of the clinical effectiveness and cost-effectiveness of opportunistic screening and stepped care interventions for older hazardous alcohol users in primary care	Opportunistic screening and stepped care intervention in primary care (20 min behavioural change counselling, motivational enhancement therapy, local specialist alcohol services)	Minimal intervention (brief advice with practice/research nurse)	CUA

a Interpreted by review authors, study authors' stated methods sometimes differed from those stated here.

### 3.1 Interventions

**Fig. 2:**
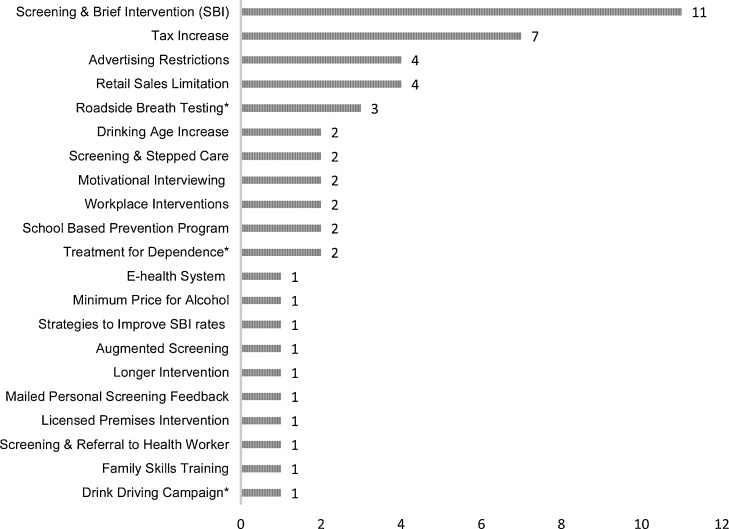
Frequency of intervention types evaluated. ^a^ The frequency of interventions is greater than the total number of included studies due to several studies considering multiple interventions. *These interventions do not fit our inclusion criteria, however they were included in studies which evaluated a mix of interventions, the rest of which did fit our inclusion criteria, therefore have been included in this figure for reference.

The most commonly evaluated intervention was screening and brief intervention (SBI) which was evaluated in 11/27 studies followed by tax increases on alcohol (n = 7). The remaining interventions evaluated ranged from targeted interventions such as family skills training and different screening variations to population level interventions such as advertising bans and raising the legal alcohol drinking age (Fig. 2).

The majority of interventions were targeted at specific “at-risk” groups and therefore in settings such as primary care (n = 13), hospital emergency departments (n = 4), hospital outpatients departments (n = 1), schools/universities (n = 3), sexual health clinics (n = 1), the community (n = 5) and the workplace (n = 2). Seven studies evaluated interventions on a national level setting (the frequency of study settings is greater than the total number of included studies due to several studies considering multiple settings).

### 3.2 Study designs

Eight randomised, and one non-randomised, controlled trials were included in the review; two of those [29], [30] considered a beyond-trial analysis longer than trial follow-up. Neighbors et al. [29] did not specify the time horizon considered but used a decision analytic model to estimate the number of quality adjusted life years (QALYs) saved for their CUA implying a time horizon longer than trial analysis (6 months) was considered; Mansdotter et al. [30] used outcomes data from a previous study to extrapolate a five year time horizon.

Eighteen modelling studies were identified; 10 of those undertook long-term evaluations which were longer than 30 years allowing for the long-term benefits of public health preventive interventions to be captured. Data were taken from the epidemiological literature, results from previous trials, longitudinal studies and meta-analyses to populate models in order to estimate future costs and outcomes.

### 3.3 Inter-sectoral costs and consequences

Ten unique cost sectors were considered (Table 3); all studies considered healthcare costs in their analyses with the exception of two studies [31], [32] that did not. The perspective taken for analysis determines the costs that will be considered since only costs relevant to that perspective are included (i.e. a study taking a healthcare perspective will only consider healthcare costs). The two studies that did not include healthcare costs conducted their evaluations according to an educational provider and societal perspective respectively. Whilst a societal perspective would traditionally include healthcare, this particular intervention was based in non-healthcare community and voluntary services; in their limitations, the authors note that healthcare resource utilisation costs were not included as would be expected in a full societal perspective.

**Table 3:** Number of studies by key study characteristics.

Study Characteristics	Studies (n)	Study numbers (Table 2)
Cost sectors		
Healthcare	25	1, 2, 3, 4, 5, 7, 8, 9, 10, 11, 13, 14, 15, 16, 17, 18, 19, 20, 21, 22, 23, 24, 25, 26, 27
Education	3	6, 17, 21
Criminal Justice	6	2, 3, 8, 19, 24, 17
Law enforcement	4	10, 11, 15, 17
Environment	0	
Employment	0	
Social care	7	3, 7, 8, 12, 20, 24, 27
Voluntary	2	3, 12
Private	2	15, 16
Out of pocket	4	5, 12, 14, 24
Government	7	4, 5, 10, 11, 14, 21, 22
Other^a^	6	2, 8, 17, 19, 20, 24
Setting		
Primary care	13	1, 2, 5, 8, 13, 14, 18, 20, 22, 23, 24, 25, 27
Hospital emergency departments	4	2, 3, 9, 19
Hospital outpatients departments	1	2
Community	5	12, 15, 23, 5, 11
National level (e.g. tax policy)	7	4, 5, 10, 11, 14, 26, 21
School/university	3	6, 17, 21
Sexual health clinics	1	7
Workplace	2	16, 21
Discounting		
Costs		
3%	13	1, 4, 5, 10, 11, 13, 14, 15, 16, 17, 19, 21, 24
3.5%	1	20
4.0%	2	25, 26
Outcomes		
3%	10	1, 4, 5, 10, 11, 13, 14, 15, 24, 17
3.5%	1	20
1.5%	2	25, 26
No discounting applied	11	2, 3, 6, 7, 8, 9, 12, 18, 22, 23, 27
Time Horizon		
<1 year	5	2, 6, 7, 8, 9
1–5 years	7	3, 12, 15, 16, 18, 23, 27
5–10 years	0	
10–30 years	2	1, 20
>30 years/Lifetime	10	5, 10, 11, 13, 14, 17, 21, 24, 25, 26
Unspecified	3	4, 19, 22
Type of study		
RCT	7	3, 6, 7, 8, 9, 12, 27
Non-randomised	1	15
Modelling study	18	1, 2, 4, 5, 10, 11, 13, 14, 16, 17, 18, 20, 21, 22, 23, 24, 25, 26
RCT + modelling	1	19
Model type		
Population model	1	14
ALCMOD	1	23
SAPM	2	20, 1
Multistate life table model	4	4, 5, 10, 11
Markov model	1	13
Decision analytic model	4	2, 18, 19, 22
Algebraic model	1	24
Chronic Disease Model	3	21, 25, 26
Statistical model	1	16
Unspecified	1	17
Stated Perspective		
Healthcare (incl. social care for NHS PSS)	11	1, 4, 5, 7, 10, 11, 20, 23, 25, 26, 27
Societal	6	3, 12, 13, 14, 15, 19
Provider	2	6, 9
Societal + Healthcare	1	24
Societal + Provider	1	2
Societal + Government	1	17
Government	1	22
Employer	1	17
Unspecified	3	8, 18, 21
Productivity costs separately accounted for		
Yes	4	2, 3, 15, 17
No	23	1, 4, 5, 6, 7, 8, 9, 10, 11, 12, 13, 14, 15, 16, 18, 19, 20, 21, 22, 23, 24, 25, 26, 27

^a^Other costs here refer to those related to automobile accidents, expenses related to property damage and insurance administration expenses.

Twelve studies stated the use of a healthcare perspective for their economic evaluation (Table 3). Of those twelve, three were conducted according to a health and personal social services perspective, therefore those studies also included costs to the social care sector. The perspective inferred from the stated costs included in the studies’ analysis differed from the stated perspective on three occasions. These three studies [33], [34], [35] additionally included costs to the government for implementing and enforcing the interventions.

Nine studies claimed to follow a societal or other broad perspective (Table 3). This perspective is recommended for interventions where benefits and costs fall outside of the healthcare sector as is often the case for public health interventions. Similarly to the studies considering a healthcare perspective, there was discrepancy between the stated and inferred perspectives in some of the studies claiming to have considered a societal perspective. Two studies considered a very narrow range of costs. The perspective used by Mansdotter et al. [30] would be better interpreted as a payer perspective since only costs related to implementing the intervention are considered. Likewise, Kapoor et al. [36] consider only healthcare and out-of-pocket patient costs which is a very narrow interpretation of the impacts to society. In addition, five of the nine studies claiming to adhere to a societal perspective did not explicitly consider productivity costs (defined as work productivity lost from illness) in their analyses, although one of those studies, Neighbors et al. [29], justified excluding estimates of productivity loss separately by stating that these are implicit in the QALY estimate. This method of accounting for productivity losses is recommended by a number of organisations such as the US Panel on cost-effectiveness analysis [9] and NICE [8], however this is not universally agreed on and those who disagree encourage specific measurement of productivity costs [37], [38].

The remaining studies conducted their analyses according to either an education, government, provider or employer perspective. The stated perspectives in these cases aligned with those inferred from the costs reported. Three studies [39], [40], [41] did not specify a perspective.

None of the studies included consequences outside of the health sector such as benefits from increased employment or to the environment. The only non-health impacts considered were cost-savings to the criminal justice sector as a result of reducing violence levels and thus avoiding resultant costs to the judicial system [30], [42].

### 3.4 Equity

None of the identified studies specifically address or discuss equity in health in their evaluations; however, inferences about effects on health inequalities may be made from data reported in some of the identified studies. Three studies [36], [43], [44] included subgroup analysis in their evaluations which stratified outcomes by gender, age and baseline alcohol consumption. Although not conducted by the authors, the Sheffield Alcohol Policy Model used by Purshouse et al. [44] allows for analysis by other variables such as socioeconomic status which could provide relevant data for an assessment of health inequalities.

## 4 Discussion

This review has confirmed the general paucity of economic evaluation studies in areas of public health that has been documented previously [1], [2], [3], [4]. Whilst this review can only comment on the status of evaluations within the arena of alcohol prevention, it would be reasonable to expect the results to be applicable to other areas of public health policy since, compared to other policy areas, alcohol is not unique in terms of economic evaluation quality. A lack of economic evaluation evidence is problematic for decision-making generally since it limits the basis for accurate, evidence-informed decisions to be made. However, there are a number of issues specific to public health decision making that have been highlighted in this review. Four are reviewed in the following sub-sections.

### 4.1 Intervention coverage

Firstly, the identified literature included evaluations of primary prevention (more upstream) interventions (e.g. tax increases or advertising bans), which impact at a whole population level, and secondary/tertiary prevention (more downstream) interventions (e.g. SBI), which target specific at-risk populations. The World Health Organisation (WHO) and the Organisation for Economic Co-operation and Development (OECD) have both promoted upstream, preventive interventions to reduce harm from alcohol (and public health generally) because of a belief in their superior cost-effectiveness [45], [46]. The literature identified in this review, however, focussed more on the evaluations of secondary/tertiary prevention interventions.

The plethora of articles evaluating interventions such as SBI may in part be due to a general acknowledgement, promoted by organisations such as the WHO and the OECD and eminent academics such as Sir Michael Marmot [47], that upstream interventions are generally cost-effective, thus inciting less perceived need to conduct such evaluations. These interventions can, however, be politically problematic to implement. Lobbying from interest groups and pressure to please voters, particularly in areas whose economy relies heavily on the food and beverages industry, prevents easy passage of policies such as alcohol tax increases or limitations on access [48], [49]. Given the politically charged context of alcohol policy decisions, economic evaluation evidence demonstrating the economic benefits, in addition to health and other benefits, could prove instrumental to the implementation of such interventions. Wanless [50] argues that health and wealth go hand-in-hand − i.e. healthy communities tend to be more economically productive − therefore if economic evaluations are also able to demonstrate positive productivity gains from upstream alcohol policies, this could provide a further counter-argument to those lobbyists opposed to such interventions.

Upstream interventions can also be more challenging to evaluate methodologically since attributing effects that occur downstream to an intervention is already often difficult. External factors will likely also affect the outcome of the intervention making it difficult to disentangle its impact. An absence of suitable data to extrapolate the downstream consequences of upstream interventions may offer another explanation for fewer economic evaluation studies of this type. Natural experiment studies can provide a means of evaluating upstream interventions [51]; for example, if policy implementation is staggered across different geographical areas and external factors can be assumed to be similar across the regions.

### 4.2 Long-term considerations

Secondly, the time-horizons adopted in some of the studies were relatively short and may not capture the impact of preventive interventions, the nature of which is to prevent uptake of unhealthy habits in the future. Long-term health risks from over-consumption of alcohol, such as various chronic diseases [52], develop over a sustained period. These health risks are unlikely to be captured in analytic time-horizons shorter than ten years. Over half of the evaluations identified had time horizons of under ten years but the critical issue is whether the inclusion of longer term effects would have changed the conclusions. Extrapolation of data for a longer horizon of analysis would have been unlikely to change the conclusion of the majority of the trial studies however at least three studies could have been strengthened from longer-term data. Crawford et al. [53] conclude that their intervention of study is not cost-effective due to no significant differences in costs or QALYs between control and intervention groups at six month follow up. Given the focus on sexual health in this study, six months may be insufficient time to expect significant differences in QALYs from participants; whereas a longer time horizon could allow for any positive effects on participants’ drinking to manifest into better sexual health, and therefore a potential increase in QALYs gained, in the long-run. Cowell et al. [32] evaluated their intervention using three month follow-up data, citing potential decline in intervention effectiveness after this point. Extrapolations would have been useful to determine whether the transitory effect of the intervention still represents value for money. Given that Cowell et al. [32] collected data up to 12 months post-intervention, excluding the longer-term data for their evaluation may also represent publication bias. A similar argument can be made for Havard et al. [54] who evaluate cost-efficacy at six weeks post-intervention. Six month follow-up is cited as underway, and has subsequently been published [55] but there has been as yet no re-estimation of cost-effectiveness based on these longer-term data.

Funding preventive interventions in public health, which mainly produce benefits that occur in the future, can be politically difficult due to an emphasis on meeting near-term targets [56]. Economic evaluations with long-term time-horizons are therefore important in order to demonstrate, with some level of certainty, the value of diverting limited funds now into programmes that, while not alleviating problems in the immediate term, may carry the kudos of achieving deferred benefits to be appreciated by someone else.

The limited proportion of studies considering the long-term consequences of interventions is predominantly driven by the economic evaluations of randomised controlled trials (RCTs). The majority of identified studies limited their analysis to a maximum of 12 months post intervention and focused on short-term outcomes which may in part be due to the cost and impracticalities of conducting RCTs with long-term follow up. Several of the studies identified in this review comment on the importance of understanding the long-term effects of the trial interventions [32], [54], [57], particularly considering potential diminishing intervention effect over time. Long-term follow-up would be required to definitively capture this; however incorporating trial results into a model alongside estimated data from literature on such external factors could enable evaluations to reflect a longer-term analysis of the intervention. This would be most useful in uncertain or costly cases, where modern modelling methods [1] could be employed to assess the potential long-run value of an intervention and aid better informed decision-making. However, it may not be necessary to consider long-run effects of all intervention trials, for example if an intervention is shown to be cost effective in the short-term and has a very low absolute cost; a more efficient use of resources could be used to fund the intervention and monitor the outcomes than devote resources to modelling.

### 4.3 Incorporating equity

Thirdly, addressing equity in health did not feature in any of the evaluation studies despite this being a globally recognised area of need [58]. This could be due to a lack of knowledge and available guidance around how to include equity considerations in economic evaluations or due to a lack of data with which to do so. If the latter were the case however, one would expect this to be highlighted by the authors in their study limitations, which is not the case in the studies identified in this review.

Methods of incorporating equity are still in relative infancy in the health economics literature, therefore it is unsurprising that this element is largely missing from the evaluations identified in this review. Recent research on methods to incorporate equity considerations into economic evaluations via CEA has been published [59], [60]. Cookson et al. [59] suggest three possible approaches to including health equity in economic evaluations: equity impact analysis, equity constraint analysis and equity weighting analysis. Equity impact analysis disaggregates relevant costs and outcomes by equity-relevant sub groups producing a dashboard of various costs and outcomes by sub group to assess who benefits and who loses from each policy option. Equity constraint analysis involves an assessment of trade-offs between the fairness (in equity terms) and cost-effectiveness of an intervention. In this analysis, equity is considered a constraint to seeking cost-effectiveness. Equity weighting analysis attempts to quantify the value of health equity impacts in order to analyse trade-offs between equity and health [61].

There is no evidence of attempts to address equity in the identified literature of this review using any of the three methods discussed. Having said this, inferences may be made from data reported in some of the studies. Some discussion points raised by authors, whilst not specifically targeting health equity, could be inferred as pertinent. For example, in discussing the impracticality of achieving the “optimal outcome” of their studied intervention, Navarro et al. [41] touch on some equality-related issues such as low adherence to the intervention by the most at-risk group of drinkers (hazardous drinkers) and lack of access to young males who are a particularly risky group.

The latter two methods outlined by Cookson et al. [59], [61], equity constraint analysis and equity weighting analysis, may be challenging to implement. However, it could be expected that an attempt at equity impact analysis be included in an evaluation so that decision-makers may consider the effects on health equity alongside efficiency.

### 4.4 Perspective

Finally, over half of the studies identified conducted their analysis according to a healthcare perspective. The Washington panel on cost-effectiveness in health and medicine proposes the use of the societal perspective in economic evaluations [9], [10] and NICE in the UK [16] recommends either a public sector or local government perspective as most appropriate for evaluations of public health interventions. NICE’s recommendation stems from acknowledgement that local government has responsibility for more than just health, therefore this should be reflected in any analysis of interventions funded by local authorities.

There are often time and resource constraints within studies which prevent a comprehensive collection of all data required to truly reflect a societal perspective [62] and some costs and benefits may be difficult to capture, such as productivity costs (discussed earlier) or benefits to family members or carers who do not directly benefit from an intervention. However, attempts should be made to include as many costs and outcomes relevant to society within the resources available; prioritising those that are likely to have the greatest impact on the outcome of the evaluation. Where precise data cannot be obtained, tools such as sensitivity analysis [63] can be used to estimate the uncertainty imposed by the missing data.

Restricting the analysis of a public health intervention to a healthcare perspective may under-value an intervention if cost-savings occur in other sectors or equally over-value the intervention if costs are incurred on other sectors as a result of the intervention. Byford and Raftery [62] argue that the societal perspective is the only perspective that is truly able to address the opportunity costs to societal welfare of the resources used for the service when the providers of the service have responsibilities beyond healthcare. Therefore, according to this argument, over half of the recent evidence available to public health decision-makers is limited to a perspective that is more restricted than their actual remit, especially given the current emphasis on addressing the wider determinants of health. However, there remains a lack of consensus over what constitutes societal welfare and thus, which costs and benefits should be included and how these should be valued. Therefore, prescribing to a societal perspective for analysis implicitly applies a judgement on what is considered to be included in the perspective which, it can be argued, is unlikely to be represent a complete picture of all socially valuable effects [64].

One of the methodological challenges identified by Weatherly et al. [3] was incorporating inter-sectoral costs and consequences. The findings from this review echo the issues raised by Weatherly and colleagues and demonstrate that this challenge has not yet been resolved. Several studies commented on difficulties incorporating certain costs and outcomes. Most limitations were around obtaining accurate costs either in general [42], [65] or specifically around non-health costs required for a full societal perspective [31], [39]. None of the studies were able to appropriately include non-health outcomes in their analyses. Identifying methods for researchers to: i) obtain appropriate data on non-health costs and consequences and ii) incorporate non-health outcomes into analyses may thus be important areas of research to pursue in future. The latter point brings with it additional limitations since the synthesis of costs and outcomes across sectors requires judgements on the related opportunity costs, i.e. what benefits are displaced elsewhere by diverting resources to a certain intervention. Considering the net effects of an intervention on the healthcare sector equivalent and directly comparable to wider society assumes that the opportunity cost of displaced resources is also equivalent amongst the various sectors. However, a unit of cost or outcome may differ in value between sectors depending on the size of the sector’s budget and the individual sector’s marginal productivity of producing a unit of outcome [66]. In order to appropriately compare inter-sectoral costs and consequences requires a value of net health benefits to be expressed equivalent to the value of net benefits to wider society [64].

An alternative approach to both adopting a societal perspective and synthesising potentially inequivalent costs and benefits could be to adopt a multi-sectoral perspective in which the net effects on each relevant sector are displayed in a disaggregated approach [64], [66]. Decision-makers can then review the merits of the intervention to each sector and make a judgement based on their own interpretation of the trade-offs in terms of costs and benefits to the relevant sectors. The CCA method would be a useful tool to represent a multi-sectoral perspective since all costs and effects are displayed in a disaggregated format for easy comparison. These could easily be displayed according to each sector. Whilst allowing for a comprehensive presentation of costs and effects, CCA alone has limitations. The disaggregated approach to presenting results in a CCA does not guide a transparent basis for decisions, through the use of cost-effectiveness thresholds for example, which can open-up the decision to scrutiny for possibly ‘cherry-picking’ results.

### 4.5 Cost-benefit analysis

A further option to incorporate multi-sector costs and benefits is through the use of CBA [66]. However, despite suggestions for greater use of CBA and CCA from organisations such as NICE, there does not appear to have been a marked increase in the number of either being published since previous reviews [2], [3]. This could be explained by a number of reasons. Firstly, NICE’s public health guidance recommending the use of CBA and CCA was only published in 2012, therefore it may be too early to see its effect filtering into published work. Secondly, NICE guidance, whilst accessible to any researcher, is most relevant to UK studies and only 5 (19%) of the studies are from UK institutions. Other countries have their own standards for economic evaluation, for instance Angus et al. [43] used QALYs because that fitted “standard practice for Italian CEA” [43]. Thirdly, a lack of available data on relevant outcomes or problems monetising outcomes, for example Cowell et al. [32] stated that CEA was chosen over CBA due to difficulty in monetizing health outcomes. Finally, the most commonly used study perspective was healthcare, therefore the adoption of a CEA or CUA approach is consistent with this. Whether considering the healthcare perspective meant that important costs and benefits were excluded, which may have altered the studies’ conclusions, is another question.

Therefore, the question that needs to be asked is whether a lack of CBA in the literature really matters. Utilising more CBA can be argued for on the grounds of wanting to provide evaluations that are a true reflection of the impact on society, as argued by Byford and Raftery [62], or to incorporate means to address/evaluate the wider determinants of health into economic evaluations which are important to public health decision-makers. The reporting of CBA results in monetary terms could be helpful when engaging in discussion with policy-makers who may be unfamiliar with health economics terminology; CBA may be more readily understood and therefore more likely to be universally accepted than a CUA, for example.

However, the potential difficulties in monetising certain benefits, such as health mentioned earlier, may complicate efforts to incorporate CBA more widely in public health evaluations. Also, many of the evaluations labelled as “cost-benefit analyses” in the literature were actually cost-analyses or CUAs [67], [68] which does not instil confidence in the execution of high quality CBA. For example, the only CBA identified by this review [69] provides no explanation of how the stated health benefits are monetised, which raises concern over the reliability of the figures used and the methodological quality of the evaluation. If quality of the study is at stake, a well-executed CUA or CEA that may not be able to address all of a decision-maker’s informational needs could be preferable to a poorly conducted CBA that attempts to meet those needs but produces an inferior evaluation.

Additionally, the identified ROI study [70] was reported as a cost-benefit analysis; however, the benefits considered were solely cost-savings and not monetised health, or other, benefits, therefore was not considered a true CBA by our definition [17]. CBA that either consider only cost-savings or very narrow benefits such as avoided fatalities (with no consideration of morbidity or quality of life) have been reported for policies and interventions that would be relevant to alcohol prevention and general public health in other sectors, such as transport economics [71], [72]. Whilst it is noteworthy that CBA is a respected method of economic evaluation in these sectors, and provides scope for its use within alcohol prevention public health, decision-makers should be mindful of whether CBA accurately reflect all the relevant costs and benefits associated with an intervention. There is clearly room for improvement on the operationalization of CBA within alcohol prevention and public heath more broadly. Further research into willingness-to-pay or discrete choice experiment studies to provide relevant data for measuring the benefits of public health interventions, would be beneficial. Until this gap in the evidence base is filled, perhaps a compromise of including a CCA, which is able to provide additional data on costs and consequences relevant to a decision-maker without the struggle of valuing and synthesising them into a single metric, may offer a potential solution.

### 4.6 Priority-setting

The general lack of published priority-setting studies in the alcohol prevention literature indicates that these methods are perhaps not yet widely considered to be useful by academics for this topic area, or that such methods are used in practice within local government or other agencies but not reported in academic or (easily accessible) grey literature. A brief search for priority-setting methods not limited to alcohol prevention did identify a small number of studies conducted in public health more broadly and a similar number within the health technology and medical fields. This finding, alongside recent research into priority setting in public health [15], [21], [73] provides scope for more incorporation of methods such as PBMA, MCDA and SROI within the area of alcohol-prevention.

Methods such as PBMA and MCDA offer decision-makers the advantage of being able to make resource allocation decisions using a systematic and transparent approach. These approaches allow for the consideration of multiple criteria, which is particularly pertinent to a public health decision-maker whose interests likely extend beyond purely population health maximisation. This is not to say, however, that pure economic evaluation methods cannot contribute to prioritization decisions. A number of the studies identified in this review demonstrated potential prioritization of interventions based on their economic evaluations. Cobiac et al. [33], as part of the Australian ACE-prevention project [74], analysed combinations of interventions to develop an “optimal intervention mix” which suggests the order in which interventions should be implemented based on their cost-effectiveness. In this example a “league table” approach is used, however the ACE-prevention study team explicitly justify the evaluative rigour in their approach to priority-setting compared to the standard league table approach.

Lai et al. [75], following guidance from the WHO-CHOICE methodology for economic evaluation (http://www.who.int/choice/en/), present their economic evaluation of multiple alcohol interventions as a cost-effectiveness frontier in which an “expansion path” can be drawn to represent the most efficient combination of interventions, indicating strategies where resources could be prioritised. The authors [75] do however note the limitations of this approach given the political and public interests which also influence policy choices in reality. The ability to address limitations such as this justifies greater use of methods such as PBMA and MCDA.

## 5 Strengths and limitations

This study reports the results of a detailed systematic review that identified literature using several databases to minimise the risk of missing data. The searches were developed with the assistance of an information specialist to ensure rigour in the search strategies. Grey literature were also examined in order to capture relevant unpublished work. Whilst attempts were made to identify relevant grey literature, this review may not have captured all of these sources, for example reports perhaps conducted by non-academic agencies that have not been externally published or disseminated. The scope of this review was limited to interventions that directly aim to reduce or prevent the misuse of alcohol and did not include interventions to prevent harm as a result of consuming alcohol. Areas such as transport economics and sexual health would likely include economic evaluations relevant to this broader scope of alcohol-induced harm and would provide a worthwhile area for exploration in future research.

Whilst the English public health context provided a focal point for the analysis of this review, the methodological findings are relevant to public health economics researchers internationally. Regardless of the setting, the inherent nature of public health interventions requires additional consideration for their evaluation, therefore the methodological challenges discussed here should be globally applicable.

## 6 Conclusions

This review echoes the findings of earlier research on the paucity of economic evaluations of public health interventions. The lack of evidence has implications for decision-making in public health, specifically due to particular methodological issues such as the types of interventions evaluated, the lack of consideration of long-term outcomes, the lack of wider societal perspectives, and lack of consideration of the impact on health inequity.

This review has also identified a gap in methods of evaluation in the published literature with very limited reporting of the use of CBA, CBA and priority-setting methods such as MCDA, PBMA, option appraisal or SROI. Given that the literature has suggested such methods of evaluation may be useful to public health decision-makers, these findings provide scope for future research into how these may be improved or adapted to aid uptake in the evaluation of public health interventions.

## Conflicts of interest

There are no conflicts of interest.

## Funding

This work was conducted as part of a PhD research project funded by Fuse, UKCRC Centre for Translational Research in Public Health which is supported with core funding from the British Heart Foundation, Cancer Research UK, Economic & Social Research Council, Medical Research Council and National Institute for Health Research.
