# Supplementary material for: Economic evaluations of alcohol prevention interventions: Is the evidence sufficient? A review of methodological challenges
Source: Health Policy. 2017 Dec;121(12):1249–62. doi: 10.1016/j.healthpol.2017.10.003 (PMC5710990; doi:10.1016/j.healthpol.2017.10.003)
Supplement: Supplementary file 1 [file mmc1.docx]

**Appendix 1 - Full search strategies used, by database**

Full search strategy for NHS EED

| **Search number** | **Search terms** |
| --- | --- |
| 1 | ((Alcohol* or drink* or intoxica* or beer or wine) ) IN NHSEED |
| 2 | MeSH DESCRIPTOR Drinking Behavior EXPLODE ALL TREES |
| 3 | MeSH DESCRIPTOR Alcohol Drinking EXPLODE ALL TREES WITH QUALIFIER PC IN NHSEED |
| 4 | MeSH DESCRIPTOR Alcoholic Beverages EXPLODE ALL TREES |
| 5 | (Drink* behavio*) IN NHSEED |
| 6 | ("Alcohol* use disorder*") OR ("alcohol* abuse") OR ("alcohol* beverage*") IN NHSEED |
| 7 | #1 OR #2 OR #3 OR #4 OR #5 OR #6 |
| 8 | (PBMA or "option appraisal" or "priority setting" or "return on investment" or ROI) IN NHSEED |
| 9 | #7 AND #8 |
| 10 | (#7) IN NHSEED FROM 2006 TO 2015 |

Search strategy for Ovid MEDLINE(R) In-Process & Other Non-Indexed Citations and Ovid MEDLINE(R) 1946 to Present

| **Search number** | **Search terms** |
| --- | --- |
| 1 | Economics/ |
| 2 | exp "costs and cost analysis"/ |
| 3 | Economics, Dental/ |
| 4 | exp economics, hospital/ |
| 5 | Economics, Medical/ |
| 6 | Economics, Nursing/ |
| 7 | Economics, Pharmaceutical/ |
| 8 | (economic$ or cost or costs or costly or costing or price or prices or pricing or pharmacoeconomic$).ti,ab. |
| 9 | (expenditure$ not energy).ti,ab. |
| 10 | value for money.ti,ab. |
| 11 | budget$.ti,ab. |
| 12 | or/1-11 |
| 13 | ((energy or oxygen) adj cost).ti,ab. |
| 14 | (metabolic adj cost).ti,ab. |
| 15 | ((energy or oxygen) adj expenditure).ti,ab. |
| 16 | or/13-15 |
| 17 | 12 not 16 |
| 18 | letter.pt. |
| 19 | editorial.pt. |
| 20 | historical article.pt. |
| 21 | or/18-20 |
| 22 | 17 not 21 |
| 23 | exp animals/ not humans/ |
| 24 | 22 not 23 |
| 25 | bmj.jn. |
| 26 | "cochrane database of systematic reviews".jn. |
| 27 | health technology assessment winchester england.jn. |
| 28 | or/25-27 |
| 29 | 24 not 28 |
| 30 | Decision Making, Organizational/mt [Methods] |
| 31 | exp Resource Allocation/mt [Methods] |
| 32 | (MCDA or PBMA).ti,ab. |
| 33 | "option appraisal".ti,ab. |
| 34 | "multi$ criteria decision analys$".ti,ab. |
| 35 | "program$ budget$ marginal analys$".ti,ab. |
| 36 | (Priority?setting adj2 method$).ti,ab. |
| 37 | "social return on investment".ti,ab. |
| 38 | (SROI or ROI).ti,ab. |
| 39 | "return on investment".ti,ab. |
| 40 | or/30-39 |
| 41 | 29 or 40 |
| 42 | (intoxica$ or beer or wine).ti,ab. |
| 43 | *drinking behavior/ |
| 44 | Alcoholic Beverages/ |
| 45 | *Binge Drinking/pc [Prevention & Control] |
| 46 | Alcohol Drinking/pc [Prevention & Control] |
| 47 | *Alcoholism/pc [Prevention & Control] |
| 48 | ("Drink$ behavio$" or "binge drink$").ti,ab. |
| 49 | (Alcohol$ adj2 ("use disorder$" or abuse or beverage$ or addiction$ or consumption or drink$)).ti,ab. |
| 50 | or/42-49 |
| 51 | 41 and 50 |
| 52 | limit 51 to (english language and humans and yr="2015 -Current") |
| 53 | remove duplicates from 52 |

Search of Embase via OVID

| **Search number** | **Search terms** |
| --- | --- |
| 1 | Health Economics/ |
| 2 | exp Economic Evaluation/ |
| 3 | exp Health Care Cost/ |
| 4 | pharmacoeconomics/ |
| 5 | 1 or 2 or 3 or 4 |
| 6 | (econom$ or cost or costs or costly or costing or price or prices or pricing or pharmacoeconomic$).ti,ab. |
| 7 | (expenditure$ not energy).ti,ab. |
| 8 | (value adj2 money).ti,ab. |
| 9 | budget$.ti,ab. |
| 10 | 6 or 7 or 8 or 9 |
| 11 | 5 or 10 |
| 12 | letter.pt. |
| 13 | editorial.pt. |
| 14 | note.pt. |
| 15 | 12 or 13 or 14 |
| 16 | 11 not 15 |
| 17 | (metabolic adj cost).ti,ab. |
| 18 | ((energy or oxygen) adj cost).ti,ab. |
| 19 | ((energy or oxygen) adj expenditure).ti,ab. |
| 20 | 17 or 18 or 19 |
| 21 | 16 not 20 |
| 22 | animal/ |
| 23 | exp animal experiment/ |
| 24 | nonhuman/ |
| 25 | (rat or rats or mouse or mice or hamster or hamsters or animal or animals or dog or dogs or cat or cats or bovine or sheep).ti,ab,sh. |
| 26 | 22 or 23 or 24 or 25 |
| 27 | exp human/ |
| 28 | human experiment/ |
| 29 | 27 or 28 |
| 30 | 26 not (26 and 29) |
| 31 | 21 not 30 |
| 32 | 0959-8146.is. |
| 33 | (1469-493X or 1366-5278).is. |
| 34 | 1756-1833.en. |
| 35 | 32 or 33 or 34 |
| 36 | 31 not 35 |
| 37 | conference abstract.pt. |
| 38 | 36 not 37 |
| 39 | *decision making/ |
| 40 | *health care planning/ |
| 41 | *resource allocation/ |
| 42 | budget/ |
| 43 | *"cost benefit analysis"/ |
| 44 | ("program$ budget$ marginal analys$" or PBMA).ti,ab. |
| 45 | ("multi?criteria decision analys$" or MCDA).ti,ab. |
| 46 | "option appraisal".ti,ab. |
| 47 | ("social return on investment" or SROI or "return on investment" or ROI).ti,ab. |
| 48 | ("Priority-setting" adj2 method$).ti,ab. |
| 49 | 39 or 40 or 41 or 42 or 43 or 44 or 45 or 46 or 47 or 48 |
| 50 | 38 or 49 |
| 51 | (intoxica$ or beer or wine).ti,ab. |
| 52 | alcohol abuse/ |
| 53 | *alcoholism/pc [Prevention] |
| 54 | *alcohol consumption/ |
| 55 | *binge drinking/pc [Prevention] |
| 56 | *drinking behavior/pc [Prevention] |
| 57 | "alcohol use disorder"/pc [Prevention] |
| 58 | ("Drink$ behavio$" or "binge drink$").ti,ab. |
| 59 | (Alcohol$ adj2 ("use disorder$" or abuse or beverage$ or addiction$ or consumption or drink$)).ti,ab. |
| 60 | 51 or 52 or 53 or 54 or 55 or 56 or 57 or 58 or 59 |
| 61 | 50 and 60 |
| 62 | limit 61 to (human and english language and yr="2015 -Current") |
| 63 | remove duplicates from 62 |

Search in PsycINFO via OVID

| **Search number** | **Search terms** |
| --- | --- |
| 1 | “Cost and cost analysis”/ |
| 2 | "Cost Containment"/ |
| 3 | (economic adj2 evaluation$).ti,ab. |
| 4 | (economic adj2 analy$).ti,ab. |
| 5 | (economic adj2 (study or studies)).ti,ab. |
| 6 | (cost adj2 evaluation$).ti,ab. |
| 7 | (cost adj2 analy$).ti,ab. |
| 8 | (cost adj2 (study or studies)).ti,ab. |
| 9 | (cost adj2 effective$).ti,ab. |
| 10 | (cost adj2 benefit$).ti,ab. |
| 11 | (cost adj2 utili$).ti,ab. |
| 12 | (cost adj2 minimi$).ti,ab. |
| 13 | (cost adj2 consequence$).ti,ab. |
| 14 | (cost adj2 comparison$).ti,ab. |
| 15 | (cost adj2 identificat$).ti,ab. |
| 16 | (pharmacoeconomic$ or pharmaco-economic$).ti,ab. |
| 17 | or/1-16 |
| 18 | (task adj2 cost$).ti,ab,id. |
| 19 | (switch$ adj2 cost$).ti,ab,id. |
| 20 | (metabolic adj cost).ti,ab,id. |
| 21 | ((energy or oxygen) adj cost).ti,ab,id. |
| 22 | ((energy or oxygen) adj expenditure).ti,ab,id. |
| 23 | or/18-22 |
| 24 | (animal or animals or rat or rats or mouse or mice or hamster or hamsters or dog or dogs or cat or cats or bovine or sheep or ovine or pig or pigs).ab,ti,id,de. |
| 25 | editorial.dt. |
| 26 | letter.dt. |
| 27 | dissertation abstract.pt. |
| 28 | or/24-27 |
| 29 | (0003-4819 or 0003-9926 or 0959-8146 or 0098-7484 or 0140-6736 or 0028-4793 or 1469-493X).is. |
| 30 | 17 not (23 or 28 or 29) |
| 31 | ("multi$ criteria decision analys$" or MCDA).ti,ab. |
| 32 | ("program$ budget$ marginal analys$" or PBMA).ti,ab. |
| 33 | ("Priority-setting" adj2 methods).ti,ab. |
| 34 | "option appraisal".ti,ab. |
| 35 | ("social return on investment" or SROI or "return on investment" or ROI).ti,ab. |
| 36 | resource allocation/ |
| 37 | *decision making/ |
| 38 | or/31-37 |
| 39 | 30 or 38 |
| 40 | alcohol abuse/ |
| 41 | binge drinking/ |
| 42 | alcohol drinking patterns/ |
| 43 | drinking behavior/ |
| 44 | alcoholic beverages/ |
| 45 | alcoholism/ |
| 46 | (intoxica$ or beer or wine).ti,ab. |
| 47 | ("Drink$ behavio$" or "binge drink$").ti,ab. |
| 48 | (Alcohol$ adj2 ("use disorder$" or abuse or beverage$ or addiction$ or consumption or drink$)).ti,ab. |
| 49 | or/40-48 |
| 50 | 39 and 49 |
| 51 | limit 50 to (human and english language and yr="2015 -Current") |
| 52 | remove duplicates from 51 |

Search in CINAHL using EBSCO

| **Search number** | **Search terms** |
| --- | --- |
| S1 | MH "Economics+" |
| S2 | MH "Financial Management+" |
| S3 | MH "Financial Support+" |
| S4 | MH "Financing, Organized+" |
| S5 | MH "Business+" |
| S6 | S2 OR S3 or S4 OR S5 |
| S7 | S1 NOT S6 |
| S8 | MH "Health Resource Allocation" |
| S9 | MH "Health Resource Utilization" |
| S10 | S8 OR S9 |
| S11 | S7 OR S10 |
| S12 | TI (cost or costs or economic* or pharmacoeconomic* or price* or pricing*) OR AB (cost or costs or economic* or pharmacoeconomic* or price* or pricing*) |
| S13 | S11 OR S12 |
| S14 | PT editorial |
| S15 | PT letter |
| S16 | PT commentary |
| S17 | S14 or S15 or S16 |
| S18 | S13 NOT S17 |
| S19 | MH "Animal Studies" |
| S20 | (ZT "doctoral dissertation") or (ZT "masters thesis") |
| S21 | S18 NOT (S19 OR S20) |
| S22 | MH “decision making” |
| S23 | MH “resource allocation” |
| S24 | TI (“multi*criteria decision analys*” or MCDA) or AB (“multi*criteria decision analys*” or MCDA) |
| S25 | TI (“program* budget* marginal analys*” or PBMA) or AB (“program* budget* marginal analys*” or PBMA) |
| S26 | TI (“priority setting” N2 method*) or AB (“priority setting” N2 method*) |
| S27 | TI (option appraisal) or AB (option appraisal) |
| S28 | TI (“social return on investment” or “return on investment” or ROI) or AB (“Social return on investment” or “return on investment” or ROI) |
| S29 | S22 OR S23 OR S24 OR S25 OR S26 OR S27 OR S28 |
| S30 | S21 OR S29 |
| S31 | MH “alcohol abuse” |
| S32 | MH “alcoholic beverages” |
| S33 | MH “drinking behavior” |
| S34 | MH “alcoholism” |
| S35 | MH “binge drinking” |
| S36 | TI (“alcohol abuse” or “alcohol misuse” or “binge drink*”) or AB (“alcohol abuse” or “alcohol misuse” or “binge drink*”) |
| S37 | TI (beer or wine or intoxica*) or AB (beer or wine or intoxica*) |
| S38 | TI (drink* behavio*) or AB (drink* behavio*) |
| S39 | TI Alcohol* N2 (“use disorder*” or beverage* or addiction* or consumption or drink*) or AB Alcohol* N2 (“use disorder*” or beverage* or addiction* or consumption or drink*) |
| S40 | S31 OR S32 OR S33 OR S34 OR S35 OR S36 or S37 or S38 or S39 |
| S41 | S30 AND S38 (with limiters: Published date: 20150101-20160531; English Language; Human) |

Search run in Scopus (health sciences and social sciences) (2006-2016)

| **Search number** | **Search terms** |
| --- | --- |
| 1 | TITLE-ABS-KEY (“return on investment” or ROI) |
| 2 | TITLE-ABS-KEY (“social return on investment” or SROI) |
| 3 | TITLE-ABS-KEY (“multi*criteria decision analys*” or MCDA) |
| 4 | TITLE-ABS-KEY (“option appraisal”) |
| 5 | TITLE-ABS-KEY (“Program* budget* marginal analys*” or PBMA) |
| 6 | TITLE-ABS-KEY ("priority setting" w/2 method*) |
| 7 | TITLE-ABS-KEY (“resource allocate*”) |
| 8 | TITLE-ABS-KEY (“multi*criteria decision aid”) |
| 9 | #1 or #2 or #3 or #4 or #5 or #6 or #7 or #8 |
| 10 | TITLE-ABS-KEY (“alcohol abuse” or “alcohol misuse” or “binge drink*”) |
| 11 | TITLE-ABS-KEY (drink* w/1 behavio*) |
| 12 | TITLE-ABS-KEY (Alcohol* w/2 (“use disorder*” or abuse or beverage* or addiction* or consumption or drink*)) |
| 13 | Or/10-12 |
| 14 | 9 AND 13 (limited to English language and year 2006-2016) |
