# Supplementary material for: Economic evaluations of alcohol prevention interventions: Is the evidence sufficient? A review of methodological challenges
Source: Health Policy. 2017 Dec;121(12):1249–62. doi: 10.1016/j.healthpol.2017.10.003 (PMC5710990; doi:10.1016/j.healthpol.2017.10.003)
Supplement: Supplementary file 2 [file mmc2.docx]

**Appendix 2 – Example data extraction form**

| General Data | | | | | | | | | | | | | | | | | | | | | | | | | | | | | | |  |
| --- | --- | --- | --- | --- | --- | --- | --- | --- | --- | --- | --- | --- | --- | --- | --- | --- | --- | --- | --- | --- | --- | --- | --- | --- | --- | --- | --- | --- | --- | --- | --- |
| Title | | | | | **Author** | | | | | **Publication year** | | | | **Publication type** | | | | | **Country of study** | | | | **Source of funding** | | | | | **Journal** | | |  |
| Cost-effectiveness of a programme of screening and brief interventions for alcohol in primary care in Italy | | | | | Angus C, Scafato E, Ghirini S, Torbica A, Ferre F, Struzzo P, Purshouse R, Brennan A | | | | | 2014 | | | | Journal article | | | | | Italy | | | | EU Framework 7 Programme | | | | | BMC Family Practice | | |  |
| Study Characteristics | | | | | | | | | | | | | | | | | | | | | | | | | | | | | | |  |
| Intervention | | | | | **Comparator(s)** | | | **Target population** | | | | | **Setting** | | | **Sample size** | | | | | | **Type of study** | | **Follow up length** | | **Time horizon** | | | **Perspective (stated)** | |  |
| Screening and brief intervention (SBI) in primary care with 2 scenarios:  1) screening at next registration  2) screening at next GP consultation | | | | | No intervention | | | Adults (over 16 years) | | | | | Primary care | | | Italian population (exact size not reported) | | | | | | Modelling (SAPM) | | 10 Years | | 30 Years | | | Healthcare (National healthcare system) | |  |
| Outcomes Data | | | | | | | | | | | | | | | | | | | | | | | | | | | | | | |  |
| Primary outcome | | | | **Secondary outcome** | | | **Tertiary outcome** | | | | | **Cost-effectiveness estimate** | | | | | | **Outcome valuation** | | | | | | | | | | | | | |
| Reduction in alcohol-attributable deaths | | | | Reduction in number of hospitalisations | | | QALYs | | | | | 1) €550/QALY next registration  2) €590/QALY next consultation | | | | | | Morbidity and mortality data taken from national databases where available. Where Italian data unavailable, UK and Dutch data used instead. No description of how QALYs valued (no mention of utility weights used). | | | | | | | | | | | | | |
| Methodological considerations | | | | | | | | | | | | | | | | | | | | | | | | | | | | | | |  |
| Extrapolation | | | | **Type of economic evaluation stated (actual)** | | | **Method of priority-setting** | | | | | **Justification of EE methods** | | | | | | | | **Equity considerations** | | | | | | | | | | |  |
| Yes. An adapted version of the Sheffield Alcohol Policy Model was used to model the full impact on health outcomes from intervention | | | | CEA (CUA) | | | N/A | | | | | None other than QALYs were used in line with standard practice for Italian cost-effectiveness analyses | | | | | | | | Outcomes reported by subgroup in terms of age, sex and mean alcohol consumption at baseline. Authors comment that data on the effectiveness of SBIs in different subgroups is still inconclusive but some evidence shows it is less effective in young adults and older drinkers. Further research is recommended into the heterogeneity of response by subgroup. | | | | | | | | | | |  |
| Reported limitations of method | | | | **Discounting** | | | **Reported strengths of method** | | | | | **Reported implications for policy/decision-making** | | | | | | | | **Details on how to use results from evaluation** | | | | | | | | | | |  |
| Main challenge reported in study was obtaining appropriate Italian data to populate model. | | | | 3% for both costs and health outcomes  (0-5% in sensitivity analysis) | | | none reported | | | | | "Cost effectiveness results provide strong recommendation for regional Italian bodies to increase use of SBIs to reduce burden on INHS" | | | | | | | | "Policy makers should be mindful of differencing cost-implications of alternative programmes of implementation. I.e. when screening occurs employs different resource distribution profiles which may affect acceptability of different SBI programme options." | | | | | | | | | | |  |
| Costs by sector | | | | | | | | | | | | | | | | | | | | | | | | | | | | | | |  |
| Healthcare | **Education** | | **Criminal Justice** | | | **Law enforcement** | | | **Environment** | | **Employment** | | | | **Social care** | | **Voluntary** | | | | **Private** | | | | **Out of pocket** | | **Government** | | | **Other** |  |
| Yes | - | | - | | | - | | | - | | - | | | | - | | - | | | | - | | | | - | | - | | | - |  |
| Productivity | | | | | | | | | | | | | | | | | | | | | | | | | | | | | | |  |
| Changes accounted for? | | **Method** | | | | | | | | | | | | | | | | | | | | | | | | | | | | |  |
| No | | - | | | | | | | | | | | | | | | | | | | | | | | | | | | | |  |
| SAPM = Sheffield Alcohol Policy Model; SBI = Screening and Brief Interventions; INHS = Italian National Health Service | | | | | | | | | | | | | | | | | | | | | | | | | | | | | | |  |
